# Epigalloccatechin-3-gallate Inhibits Ocular Neovascularization and Vascular Permeability in Human Retinal Pigment Epithelial and Human Retinal Microvascular Endothelial Cells via Suppression of MMP-9 and VEGF Activation

**DOI:** 10.3390/molecules190812150

**Published:** 2014-08-13

**Authors:** Hak Sung Lee, Jae-Hyun Jun, Eun-Ha Jung, Bon Am Koo, Yeong Shik Kim

**Affiliations:** 1 Natural Products Research Institute, College of Pharmacy, Seoul National University, 1 Gwanak-ro, Gwanak-gu, Seoul 151-742, Korea; E-Mail: mildpeople@snu.ac.kr; 2 Research Center, Samil Pharmaceutical Co. Ltd., 216 Sandan-ro, Danwon-gu, Ansan 425-852, Korea; E-Mails: wogustodrkr@nate.com (J.-H.J.); eunha.jung@samil-pharm.com (E.-H.J.)

**Keywords:** EGCG, ARPE-19, HRMEC, ocular neovascularization, vascular permeability, MMP-9, VEGF

## Abstract

Epigalloccatechin-3-gallate (EGCG) is the main polyphenol component of green tea (leaves of *Camellia sinensis*). EGCG is known for its antioxidant, anti-inflammatory, antiviral, and anti-carcinogenic properties. Here, we identify EGCG as a new inhibitor of ocular angiogenesis and its vascular permeability. Matrix metalloproteinases (MMPs) and vascular endothelial growth factor (VEGF) play a key role in the processes of extracellular matrix (ECM) remodeling and microvascular permeability during angiogenesis. We investigated the inhibitory effects of EGCG on ocular neovascularization and vascular permeability using the retina oriented cells and animal models induced by VEGF and alkaline burn. EGCG treatment significantly decreased mRNA and protein expression levels of MMP-9 in the presence of 12-O-tetradecanoylphorbol-13-acetate (TPA) and tumor necrosis factor alpha (TNF-α) in human retinal pigment epithelial cells (HRPECs). EGCG also effectively protected ARPE-19 cells from cell death and attenuated mRNA expressions of key angiogenic factors (MMP-9, VEGF, VEGF Receptor-2) by inhibiting generation of reactive oxygen species (ROS). EGCG significantly inhibited proliferation, vascular permeability, and tube formation in VEGF-induced human retinal microvascular endothelial cells (HRMECs). Furthermore, EGCG significantly reduced vascular leakage and permeability by blood-retinal barrier breakdown in VEGF-induced animal models. In addition, EGCG effectively limited upregulation of MMP-9 and platelet endothelial cell adhesion molecule (PECAM/CD31) on corneal neovascularization (CNV) induced by alkaline burn. Our data suggest that MMP-9 and VEGF are key therapeutic targets of EGCG for treatment and prevention of ocular angiogenic diseases such as age-related macular degeneration, diabetic retinopathy, and corneal neovascularization.

## 1. Introduction

Neovascularization is the process of formation of new capillary vessels from preexisting blood vessels. Abnormal angiogenesis is a leading cause of many ocular diseases such as diabetic retinopathy (DR), age-related macular degeneration (AMD), and corneal neovascularization (CNV) [1,2,3,4]. Previous studies have reported that angiogenic events are regulated by several growth factors, such as vascular endothelial growth factor (VEGF), basic fibroblast growth factor (bFGF), and platelet-derived growth factor (PDGF) [5,6,7]. Most studies on early pathogenesis of AMD have focused on oxidative injuries affecting the retinal pigment epithelium (RPE). In fact, cumulative oxidant injury to the RPE plays an important role in development of AMD [8,9]. In addition, late development of AMD demonstrates accumulation of specific deposits and extracellular matrix (ECM) molecules under the RPE. It is characterized by early degradation of the ECM followed by proliferation and migration of the endothelial cells [10]. One of the early events in DR is the breakdown of the blood–retinal barrier (BRB), leading to the leakage of blood vessels, resulting in retinal disorders such as diabetic macular edema. The BRB consists of an outer component, the retinal pigment epithelial cells, an inner component, and the retinal vascular endothelial cells [11]. The BRB plays a central role in maintaining the homeostasis of the retinal microenvironment for suppressing the diabetic retina complications [12]. In corneal neovascularization, new capillary vessels arise from the pericorneal plexus and sprout into the stroma [13,14]. Key mediators such as VEGF, bFGF, and matrix metalloproteinases (MMPs) are known to play an important role in this process [15]. MMPs secreted by endothelial cells are hypothesized to play a key role in the processes of ECM remodeling during angiogenesis [16,17]. MMP-2 and MMP-9 are extracellular proteinases that have been revealed to play an important role in retinal neovascularization and increasing of vascular permeability seen in the late development of diabetic retinopathy [18,19]. Especially, MMP-9 is essential for stable basement membrane organization in repair by CNV [20]. Physiological balance of vascular biomarkers such as VEGF and pigment epithelium derived factor (PEDF) is crucial to maintain normal vasculature [21]. The imbalance of these angiogenic factors and related proteins may lead to sequestered pro-angiogenic events. Angiogenesis consists of a sequential process that includes digestion of the ECM barrier underlying the blood vessel endothelial cell layers by MMPs, and migration and proliferation of endothelial cells [22].

**Figure 1:**
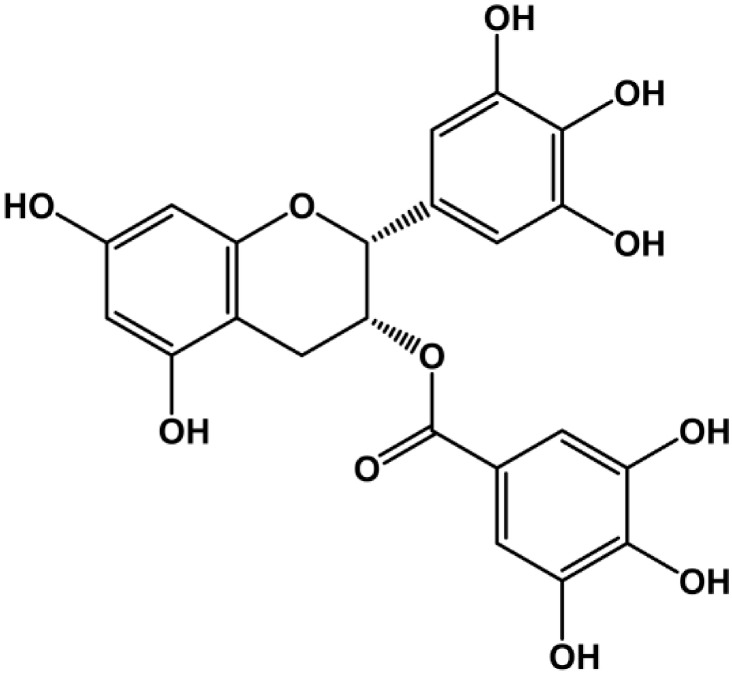
Chemical structure of EGCG.

Epigallocatechin-3-gallate (EGCG) is the most abundant polyphenol of green tea (leaves of *Camellia sinensis*) (Figure 1). Several catechin derivatives found in green tea extract exhibit antioxidant, anti-inflammatory, anti-carcinogenic, and antiviral properties [23,24]. Especially, EGCG has been studied extensively as a potential treatment for a variety of oxidative stress related diseases including cancer and cardiovascular and degenerative diseases due to its capacity to inhibit a variety of inflammatory and angiogenic factors such as tumor necrosis factor α (TNF-α) and VEGF [25,26]. Its angiostatic potential was suggested by its inhibition of new blood vessel formation in a chick chorioallantoic membrane model, and suppression of oxygen-induced retinal neovascularization and corneal neovascularization in animal models [27,28,29]. EGCG was found to decrease VEGF receptor phosphorylation in human umbilical arterial endothelial cells (HUAECs) and has also been reported to inhibit the MMP-2 and MMP-9 in human umbilical vein endothelial cells (HUVECs) [30,31]. 

Whether these angiogenic factors, including VEGF and ECM-related molecules, play an important role in RPE and retinal microvascular endothelial (RME) cells, and the precise mechanism by which they do so, is unclear. In the present study, we propose that EGCG can protect RPE and RME cells from ROS-induced BRB remodeling and angiogenesis through its antioxidative and anti-angiogenic effects. We show for the first time that EGCG protects human retina cells against BRB alteration (breakdown and remodeling), ocular neovascularization, and vascular permeability via suppression of MMP-9 and the consequent VEGF activation in RPE and RME cells.

## 2. Results and Discussion 

### 2.1. EGCG Has Less Cytotoxicity in Retinal Pigment Epithelial Cells and Retinal Microvascular Endothelial Cells

**Figure 2:**
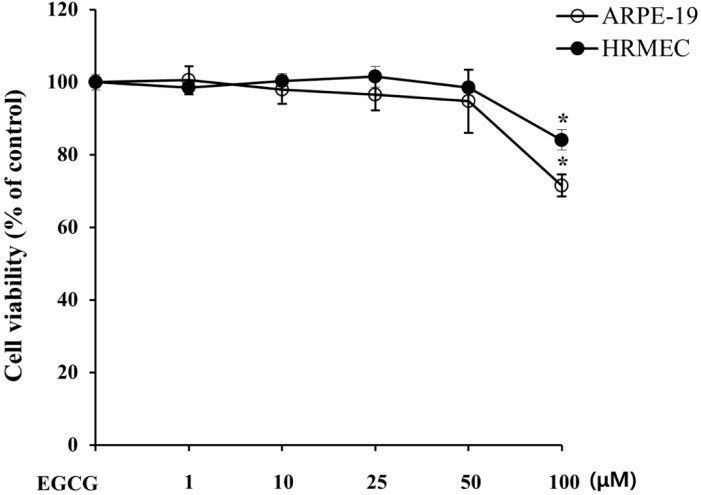
Cytotoxicity of EGCG to ARPE-19 and HRMEC. ARPE-19 cells and HRMEC were treated with or without EGCG for 24 h, and cell viability was assessed by 3-(4,5-dimethylthiazol-2-yl)-2,5-diphenyltetrazolium bromide (MTT) assay. The results are expressed as percentage of control and are presented by mean ± SD (n = 9). *, *p* < 0.01 *vs.* EGCG untreated.

To examine whether EGCG induces a cytotoxic effect in RPE and RME cells, we evaluated cell viability at doses ranging from 1 to 100 μM EGCG. As shown in Figure 2, EGCG treatment did not significantly affect cell viability up to 50 μM. Therefore, 1–50 μM of EGCG were used for the subsequent cell-based *in vitro* experiments.

### 2.2. EGCG Inhibits TPA-Induced MMP-9 Protein Production and mRNA Expression in ARPE-19 Cells 

**Figure 3:**
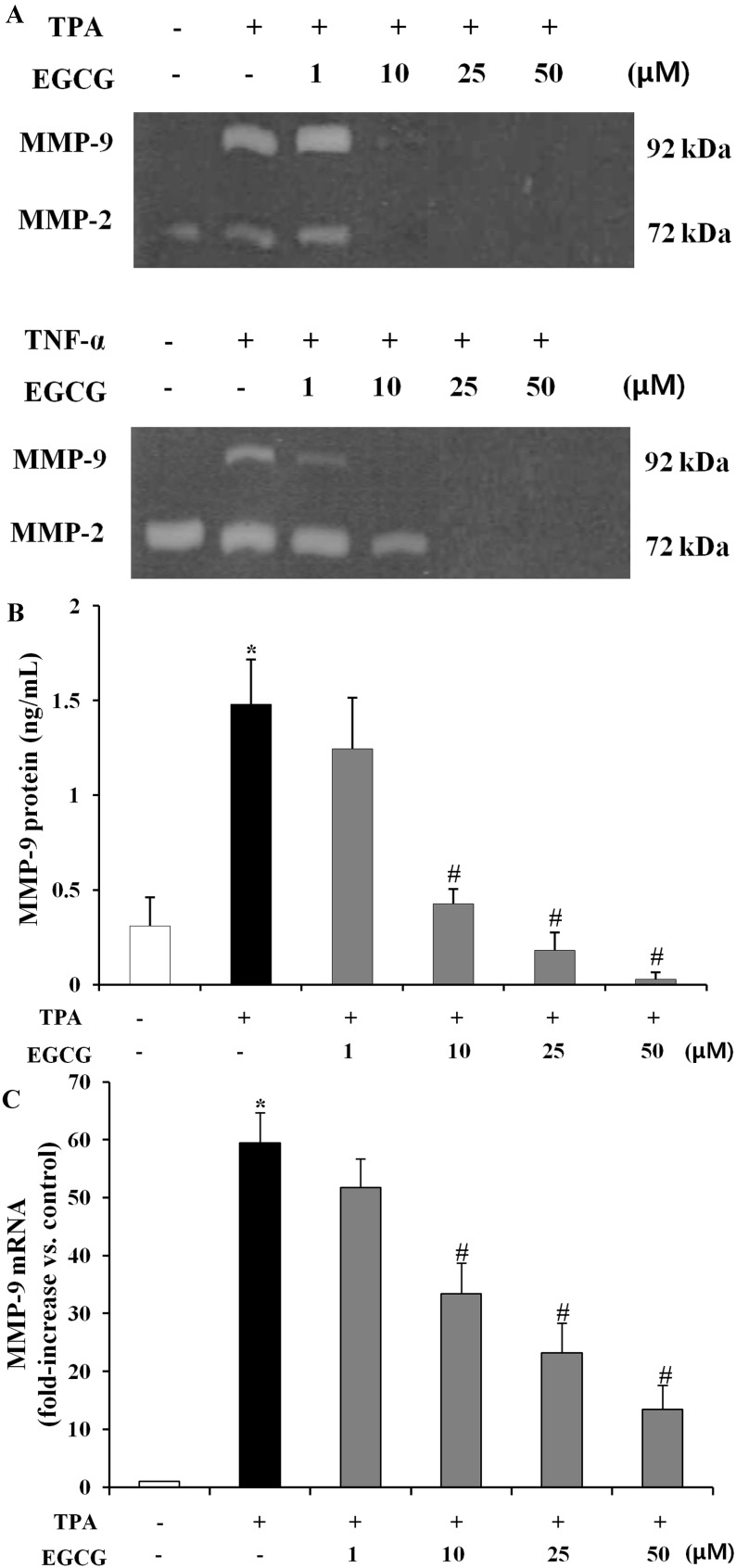
Characterization of MMP-9 in ARPE-19 cells treated with EGCG. (**A**) Gelatin zymography was performed using ARPE-19 cell lysates treated with 10 ng/mL TPA, 10 ng/mL TNF-α, and 1–50 μM EGCG in serum-free medium for 24 h. Figures were selected as representative data from three independent experiments. The positions of MMP-2 and MMP-9 are indicated; (**B**) MMP-9 protein production at 24 h after TPA or EGCG treatment was determined by ELISA. The results are presented by mean ± SD (n = 9). *, *p* < 0.01 *vs*. TPA and EGCG untreated; #, *p* < 0.01 *vs*. TPA only; (**C**) MMP-9 mRNA expression levels at 24 h after TPA or EGCG treatment was analyzed by quantitative real-time PCR. The level of MMP-9 mRNA, corrected for differences in GAPDH levels between samples, are represented as fold induction of control and are presented by mean ± SD (n = 9). *, *p* < 0.01 *vs*. TPA and EGCG untreated; #, *p* < 0.01 *vs*. TPA only.

MMPs and their tissue inhibitors (TIMPs) play an important role in the pathogenesis in AMD [32]. In particular, formation of choroidal neovascular membrane in AMD is closely associated with an accumulation of MMP-2 and MMP-9 [33]. To evaluate the inhibitory effect of EGCG on MMPs expression in human retinal epithelial cells, we examined whether EGCG might inhibit TPA and TNF-α induced MMP (MMP-2 and MMP-9) expression using the gelatin zymography, enzyme-linked immunosorbent assay (ELISA) and quantitative real-time fluorescence polymerase chain reaction (qRT-PCR) [34]. As shown in Figure 3A, MMP-9 activity was significantly suppressed by co-treatment with TPA (10 ng/mL) or TNF-α (10 ng/mL) and EGCG (1–50 μM). However, the MMP-2 level showed no difference between the groups by TPA or TNF-α induction in ARPE-19 cells, also mRNA expression level of MMP-2 showed no significant difference compared with the EGCG treatment group (data not shown). But the inhibitory effects of EGCG on MMP-2 activity and its regulatory molecules were studied in human breast cancer cell line (MCF-7) [35]. Next, we measured MMP-9 protein and mRNA expression level with EGCG (1–50 μM) in ARPE-19 cells. As shown in Figure 3B, MMP-9 protein was significantly elevated (4.78-fold, *p* < 0.01) by TPA, which was, however, dramatically reduced (0.71- to 0.98- fold, *p* < 0.01) by EGCG (10–50 μM) treatment. Additionally, MMP-9 mRNA level by co-treatment with TPA (10 ng/mL) and EGCG (10–50 μM) was found to have a decrease (0.50- to 0.71-fold, *p* < 0.01) in the amount of mRNA in the TPA-induced control (Figure 3C).

### 2.3. Inhibition of H_2_O_2_-Induced Cell Death and ROS Production by EGCG in ARPE-19 Cells

**Figure 4:**
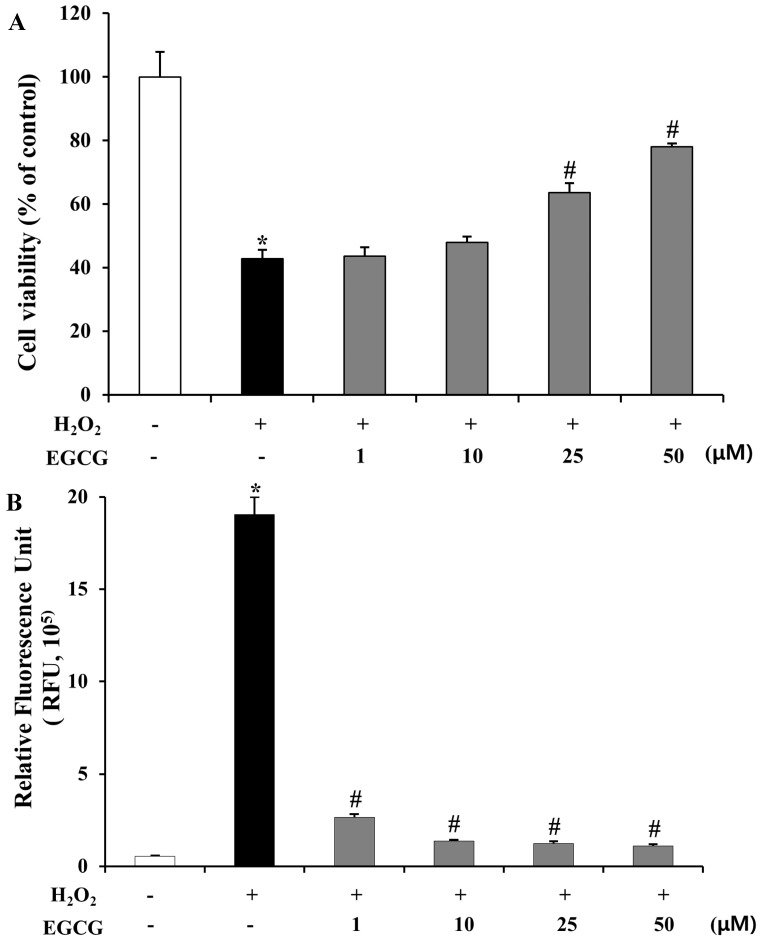
Effects of EGCG on H_2_O_2_-induced cell death and ROS production in ARPE-19 cells. **(A)** Cell viability was assessed in ARPE-19 cells treated with 600 μM H_2_O_2_ or EGCG (1–50 μM) for 24 h by MTT assay. The results are expressed as percentage of control and are presented by mean ± SD (n = 9). *, *p* < 0.01 *vs.* H_2_O_2_ and EGCG untreated; #, *p* < 0.01 *vs*. H_2_O_2_ only; (**B)** ARPE-19 cells were pretreated with EGCG (1–50 μM) for 0.5 h and then treated with 600 μM H_2_O_2_ for 15 min. For measuring H_2_O_2_ production, the cells were then labeled with DCFH-DA. Quantitative analysis was performed by measuring the fluorescence intensity. The results are presented by mean ± SD (n = 9). *, *p* < 0.01 *vs*. H_2_O_2_ and EGCG untreated; #, *p* < 0.01 *vs*. H_2_O_2_ only.

Reactive oxygen species are known to induce apoptosis and necrosis [36,37]. In a previous study, EGCG did not significantly induce apoptosis or necrosis with up to 100 μM in ARPE-19 cells [38]. To investigate the inhibitory effects of EGCG on cell death and ROS production in human retinal epithelial cells, we measured cell viability and intracellular ROS activity using the MTT and cell-permeable fluorescence dye (DCFH-DA). As shown in Figure 4A, cell viability of ARPE-19 cells was decreased (57%, *p* < 0.01) after H_2_O_2_ (600 μM) exposure. However, EGCG (25 and 50 μM) treatment effectively protected (63.6%–78.1%, *p* < 0.01) ARPE-19 cells from H_2_O_2_-induced cell death. Next, we measured intracellular ROS with different concentrations of EGCG (1–50 μM) in H_2_O_2_-induced ARPE-19 cells. As shown in Figure 4B, ROS generation was significantly increased by H_2_O_2_ (34.4-fold, *p* < 0.01), which was, however, dramatically reduced (0.86- to 0.94-fold, *p* < 0.01) by EGCG (1–50 μM) treatment. 

### 2.4. EGCG Suppresses Expression of MMP-9, VEGF, and VEGFR-2 on H_2_O_2_-Induced Oxidative Stress in ARPE-19 Cells 

**Figure 5:**
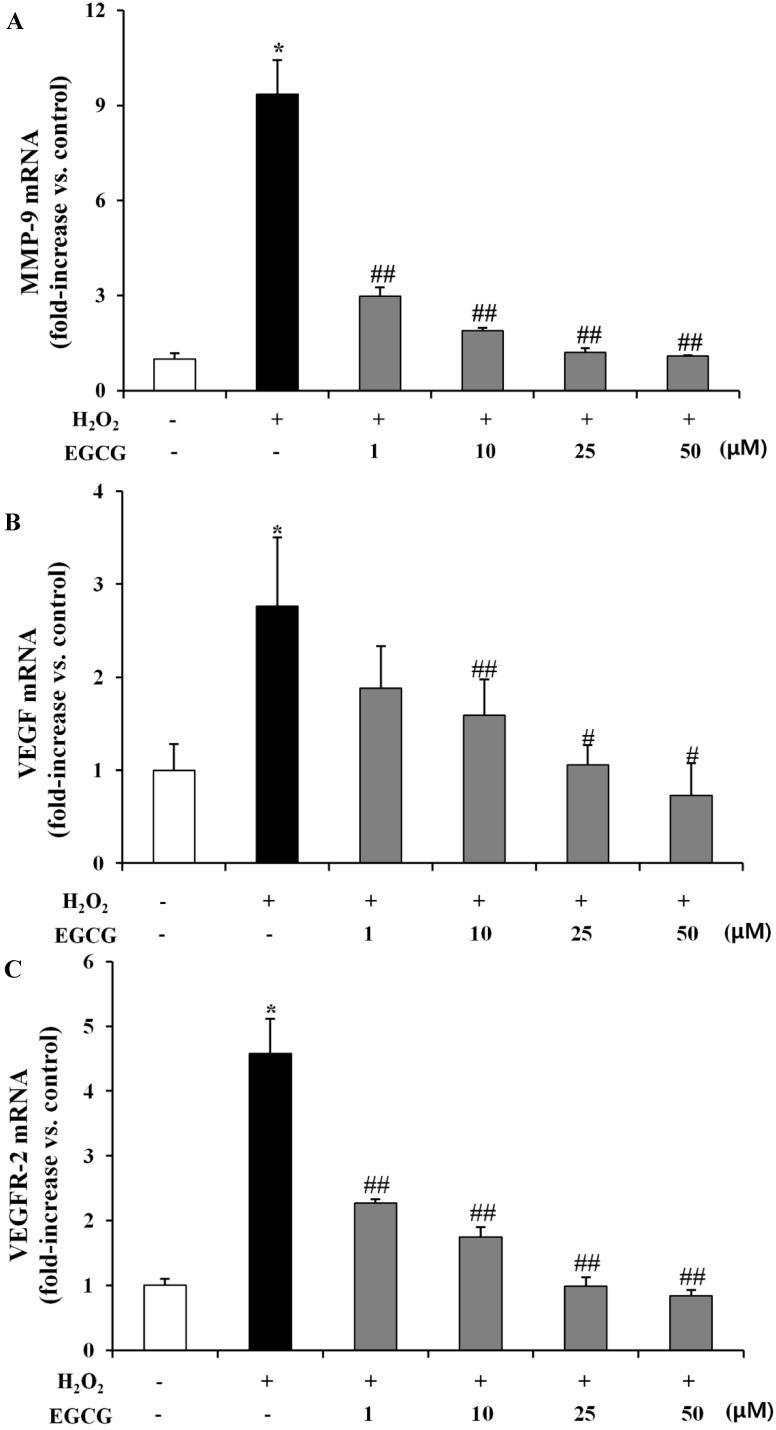
EGCG suppresses expression of MMP-9, VEGF, and VEGFR-2 on H_2_O_2_-induced oxidative stress in ARPE-19 cells MMP-9 (**A**), VEGF (**B**), VEGFR-2 (**C**) mRNA expression levels at 24 h after H_2_O_2_ (300 μM) or EGCG (1–50 μM) treatment was analyzed by quantitative real-time PCR. The expression levels of mRNA, corrected for differences in GAPDH levels between samples, are represented as fold induction of control and are presented by mean ± SD (n = 9). *, *p* < 0.01 *vs*. H_2_O_2_ and EGCG untreated; #, *p* < 0.01 *vs*. H_2_O_2_ only; ##, *p* < 0.05 *vs*. H_2_O_2_ only.

VEGF and its receptors are known to be the main modulators of angiogenesis, in which VEGF interacts with receptor tyrosine kinase (VEGFR-1 and VEGFR-2) [25,39]. In addition, MMPs play a key role in the processes of ECM remodeling during angiogenesis [17]. EGCG can remove VEGF-induced signaling by interfering with the formation of a receptor complex in HUVECs [40]. To investigate the inhibitory effects of EGCG on MMP-9, VEGF, and VEGFR-2 expression by H_2_O_2_-induced oxidative stress in human retinal epithelial cells, we examined whether EGCG might inhibit H_2_O_2_ (300 μM) induced MMP-9, VEGF, and VEGFR-2 expression using the qRT-PCR. As shown in Figure 5, in the H_2_O_2_-alone group MMP-9, VEGF and VEGFR-2 levels were increased by 9.35-, 2.76- and 4.58-fold, respectively, compared to the normal group (*p* < 0.01 for MMP-9, *p* < 0.01 for VEGF, *p* < 0.05 for VEGFR-2). However, treatment with EGCG showed lower mRNA expression of MMP-9 (0.68- to 0.88-fold, *p* < 0.05) (Figure 5A), VEGF (0.42- to 0.61-fold, *p* < 0.05 for 10–50 μM EGCG) (Figure 5B) and VEGFR-2 (0.51- to 0.82-fold, *p* < 0.05) (Figure 5C) by EGCG (1–50 μM) treatment, respectively, relative to the H_2_O_2_-alone group. VEGFR-1 signal could not be quantified in the ARPE-19 cells by qRT-PCR, probably because of very low levels of expression (data not shown).

### 2.5. EGCG Inhibits VEGF-Induced Proliferation and Vascular Permeability in HRMECs

**Figure 6:**
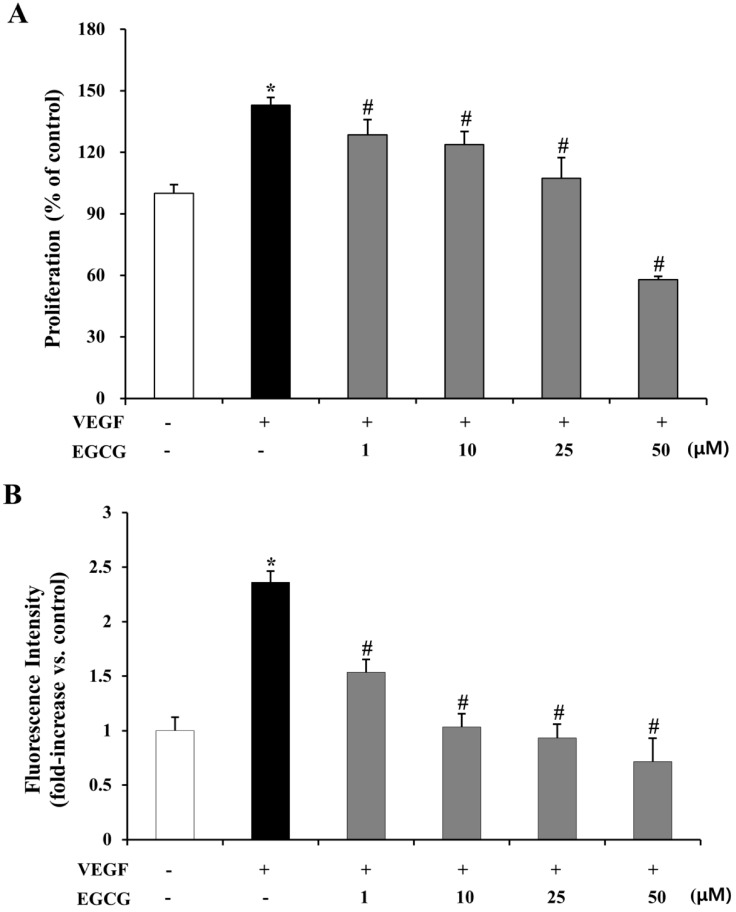
Inhibitory effect of EGCG on VEGF-induced proliferation and vascular permeability by HRMECs. (**A**) HRMECs were treated with 10 ng/mL VEGF or 1–50 μM EGCG for 72 h. Cell viability was assessed by MTT assay. The results are expressed as percentage of control and are presented by mean ± SD (n = 9). *, *p* < 0.01 *vs*. VEGF and EGCG untreated; #, *p* < 0.01 *vs*. VEGF only; (**B**) HRMECs were treated with 10 ng/mL VEGF or 1–50 μM EGCG for 72 h. Permeability was analyzed by measuring the fluorescence of FITC-labeled dextran across the monolayer cultured HRMECs. The results are expressed as fold induction of control and are presented by mean ± SD (n = 9). *, *p* < 0.01 *vs*. VEGF and EGCG untreated; #, *p* < 0.01 *vs*. VEGF only.

VEGF may induce various angiogenic activities, including the endogenous upregulation of vessel permeability of endothelial cells [41,42]. To evaluate the inhibitory effect of EGCG on VEGF-induced proliferation of human retinal endothelial cells, we measured cell viability and the fluorescence of FITC-labeled dextran across the monolayer using the MTT and vascular permeability assay in HRMECs. As shown in Figure 6A, in the VEGF alone group, the proliferation of HRMECs was increased (43.0%, *p* < 0.01) compared with the control. Proliferative activity was significantly suppressed (10.0%–59.4%, *p* < 0.01) by co-treatment with VEGF (10 ng/mL) and EGCG (1–50 μM). Next, we measured FITC-dextran permeability with different concentrations of EGCG (1–50 μM) in VEGF-induced HRMECs. As shown in Figure 6B, FITC-dextran permeability was significantly increased by VEGF (2.36-fold, *p* < 0.01), which was, however, effectively reduced (0.35- to 0.70-fold, *p* < 0.01) by EGCG (1–50 μM) treatment.

### 2.6. Inhibition of VEGF-Induced Tube Formation by EGCG in HRMECs

**Figure 7:**
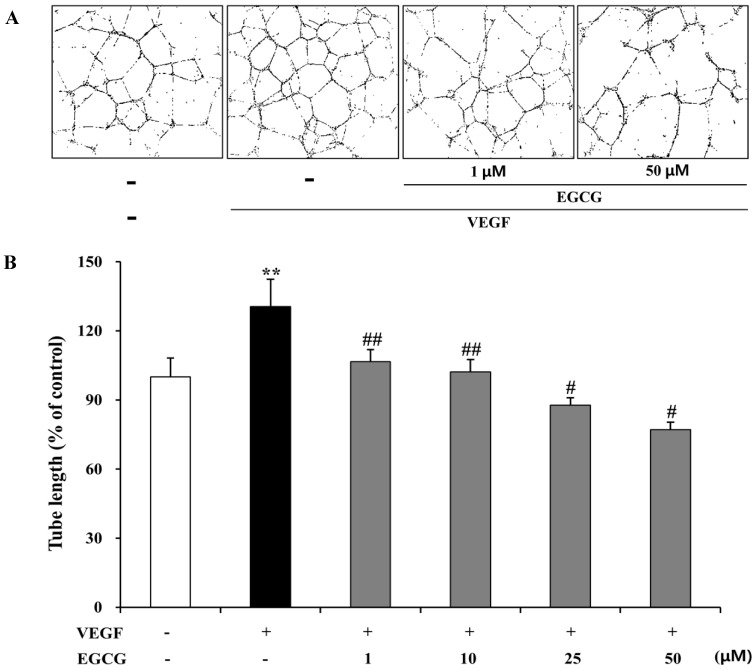
Inhibitory effect of EGCG on tube formation by HRMECs. (**A**) HRMECs were inoculated on the surface of the basement membrane matrix and treated with 10 ng/mL VEGF or 1–50 μM EGCG for 24 h. Figures were selected as representative photographs from three independent experiments; (**B**) Tube formation was observed in three randomly chosen fields, and the basal tube length of HRMECs without VEGF and EGCG (1–50 μM) was normalized to 100%. Tube length was measured using an image analyzer. The results are expressed as percentage of control and are presented by mean ± SD (n = 9). **, *p* < 0.05 *vs*. VEGF and EGCG untreated; #, *p* < 0.01 *vs*. VEGF only; ##, *p* < 0.05 *vs*. VEGF only.

To investigate the inhibitory effects of EGCG on VEGF-induced tube network formation in human retinal endothelial cells, we evaluated whether EGCG could inhibit VEGF-induced tube formation in HRMECs. As shown in Figure 7A, the formation of capillary-like structures by HRMECs was significantly suppressed by co-treatment with VEGF (10 ng/mL) and EGCG (1 and 50 μM). As shown in Figure 7B, tube formation (length, %) was significantly increased by VEGF (30.5%, *p* < 0.05), which was, however, significantly suppressed (23.9- to 53.5-%, *p* < 0.05) by EGCG (1–50 μM) treatment. The tube formation by HUVECs was significantly suppressed by EGCG (1–50 μM, *p* < 0.05) treatment (data not shown). 

### 2.7. EGCG Inhibits VEGF-Induced Vascular Permeability in Vivo

**Figure 8:**
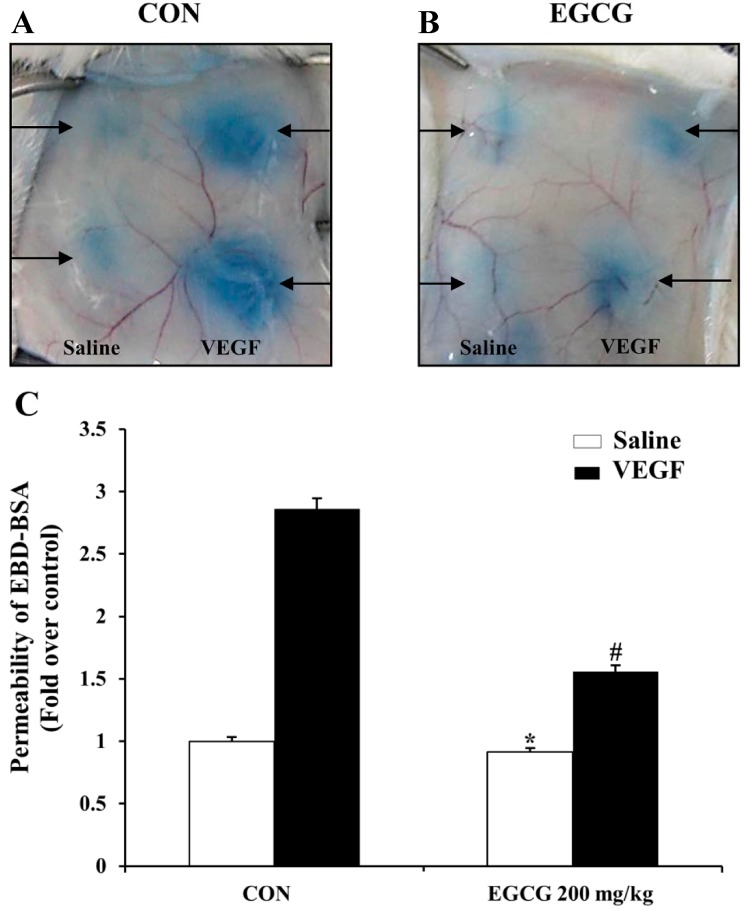
EGCG inhibits VEGF-induced vascular permeability *in vivo*. (**A**) and (**B**) VEGF induced vascular leakage of serum albumin-bound Evans Blue in the back skin of mice pretreated orally with EGCG (200 mg/kg) or saline for 1.5 h. Figures were selected as representative photograph from three independent experiments. (**C**) Spectrophotometric analysis of vascular leakiness by formamide-extracted Evans Blue dye content. Evans Blue dye content of skin injected with both of VEGF (50 ng) and EGCG (200 mg/kg) was significantly decreased compared with that of skin injected with VEGF (50 ng) alone. The results are expressed as fold induction of control and are presented by mean ± SD (n = 6 mice per group). *, NS *vs*. saline treated in control group; #, *p* < 0.01 *vs*. VEGF treated in control group.

To establish the protective effect of EGCG on vascular endothelial permeability *in vivo*, we evaluated EGCG in the mouse model of vascular leakage [43]. As shown in Figure 8A,B, vascular permeability in the back skin of mice pretreated orally with EGCG (200 mg/kg) was effectively decreased compared with the control (VEGF alone). As shown in Figure 8C, vascular leakage (content of albumin-bound Evans Blue dye) was significantly increased by VEGF (2.86-fold, *p* < 0.01), which was, however, significantly suppressed (0.46-fold, *p* < 0.01) by EGCG (200 mg/kg) treatment.

### 2.8. EGCG Inhibits VEGF-Induced Vascular Leakage by Blood-Retinal Barrier Breakdown in Vivo

**Figure 9:**
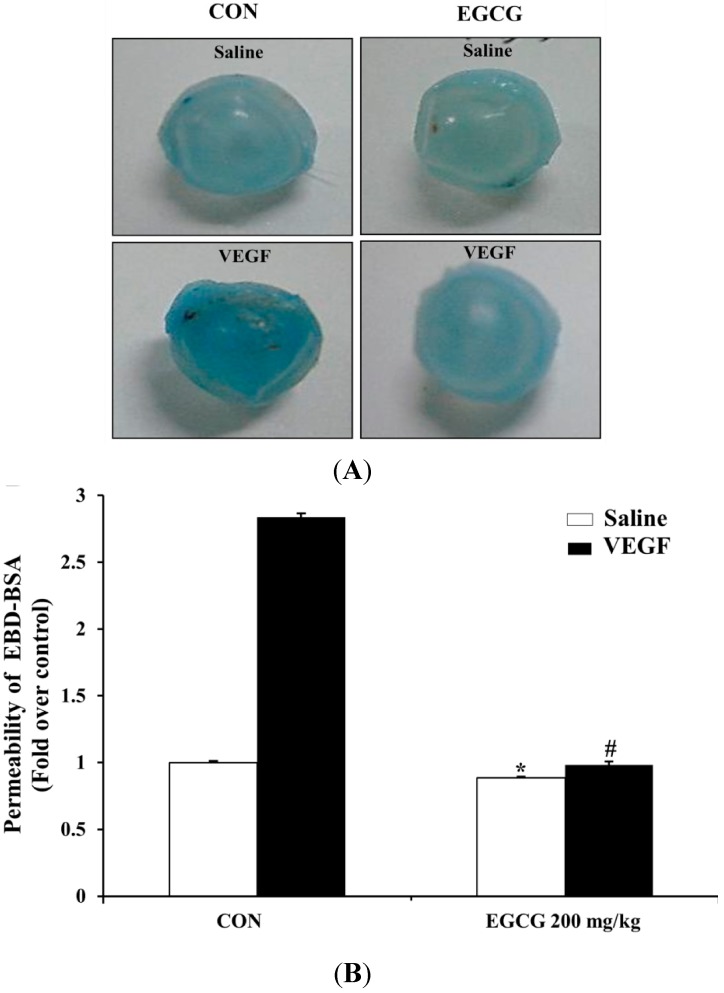
EGCG inhibits VEGF-induced vascular leakage by blood-retinal barrier breakdown *in vivo*. (**A**) VEGF induced vascular leakage of serum albumin-bound Evans Blue in the eyeball of rat administered orally with EGCG (200 mg/kg) or saline for 4 days. Figures were selected as representative photographs from three independent experiments; (**B**) Spectrophotometric analysis of vascular leakiness by formamide-extracted Evans Blue dye content. Evans Blue dye content of eyes injected intravitreally with VEGF (50 ng) after EGCG (200 mg/kg) orally pretreatment was significantly decreased compared with that of eyes injected with VEGF alone. The results are expressed as fold induction of control and are presented by mean ± SD (n = 6 mice per group). *, NS *vs*. saline treated in control group; #, *p* < 0.01 *vs*. VEGF treated in control group.

To evaluate the inhibitory effect of EGCG on retinal vasopermeability by blood-retinal barrier breakdown in vivo, we evaluated EGCG using the rat model of blood-retinal barrier permeability [44]. As shown in Figure 9A, vascular permeability in the eyeball of rat pretreated orally with EGCG (200 mg/kg) for 4 days was effectively suppressed compared with control (VEGF alone). As shown in Figure 9B, vascular permeability was significantly increased by VEGF (2.83-fold, *p* < 0.01), which was, however, significantly suppressed (0.65-fold, *p* < 0.01) by EGCG (200 mg/kg) treatment.

### 2.9. EGCG Inhibits Corneal Neovascularization via the Suppression of MMP-9 Activity in an Alkali Burn-Induced Model of Corneal Angiogenesis in Vivo

**Figure 10:**
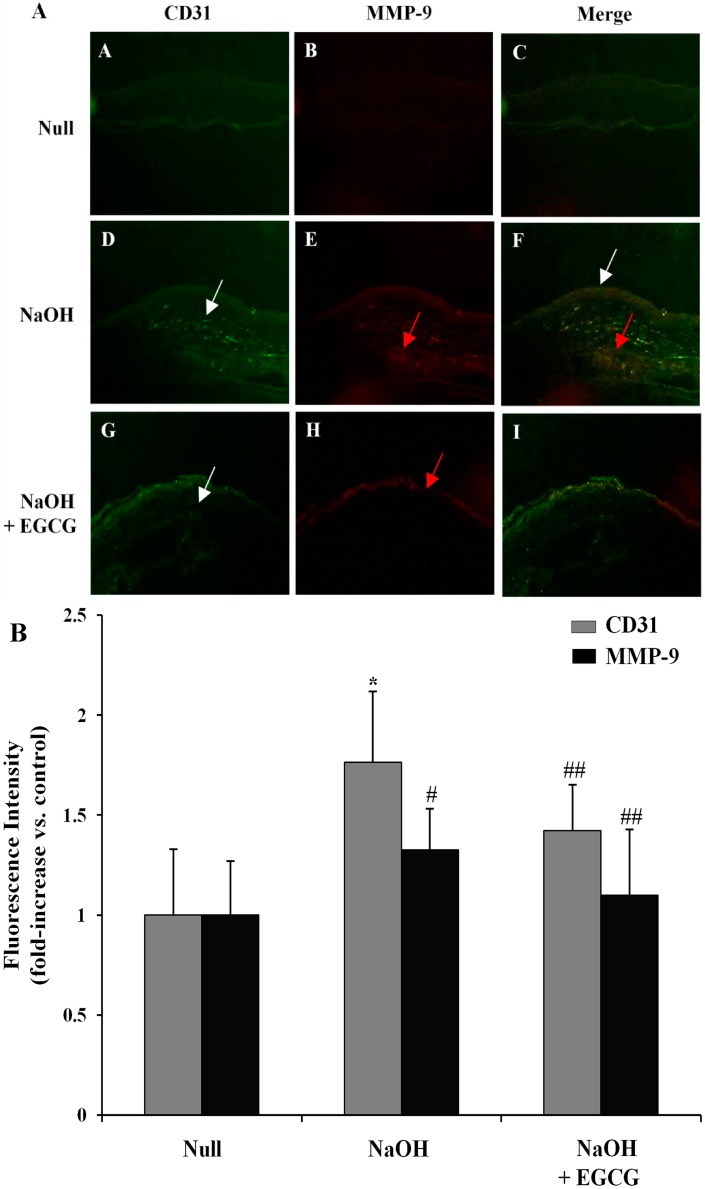
EGCG inhibits expression of MMP-9 and CD31 (PECAM-1) on corneal neovascularization (CNV) induced by alkaline burn. **(A)** Representative cornea images captured with a fluorescence microscope on day 3 after alkali burns treated with subconjunctival injection of EGCG (50 μM, 2 μL) or saline. Fluorescent immunohisto-chemistry shows that CD31 (Green Fluor, A, D, G; magnification 100×) and MMP-9 (Red Fluor, B, E, H; magnification 100×) are highly expressed in the control (NaOH only) cornea. White arrows indicate corneal epithelium and red arrows are stroma. Figures were selected as representative photographs from four independent experiments; (**B)** Fluorescence intensities were analyzed using a NIH Image 1.45 program. The results are expressed as fold induction of control and are presented by mean ± SD (n = 6 mice per group). *, *p* < 0.01 *vs*. Null; #, *p* < 0.05 *vs*. Null; ##, *p* < 0.05 *vs*. NaOH only.

Chemical burn-induced CNV is closely related to release of inflammatory cytokines and MMPs for corneal angiogenesis and epithelial breakdown [45]. In this process, MMPs participate significantly in the degradation of the vascular basement membrane and remodelling of the ECM for the formation and development of CNV [46,47]. To examine the inhibitory effects of EGCG on alkali burn-induced corneal angiogenesis in rat cornea, we evaluated whether EGCG could suppresses corneal stromal neovascularization on day 3, treated with subconjunctival injection of EGCG (50 μM, 2 μL) or saline after alkali burns (0.5 N NaOH for 60 s). As shown in Figure 10A, CD31 (PECAM-1) positive cells express Green Fluor. Corneal neovascularization occurs around the epithelium (white arrow) of the cornea on day 3 post-burn, and the stromal cells express MMP-9 (Red Fluor) at the peripheral cornea (red arrow) on day 3 (A-I; magnification 100×). As shown in Figure 10B, double-labeling fluorescent intensity showed that CD31 and MMP-9 were effectively upregulated in alkali burn-induced corneas compared with normal corneas (1.76-fold for CD31, *p* < 0.01, and 1.32-fold for MMP-9, *p* < 0.05), which was, however, significantly suppressed (0.19-fold for CD31 and 0.17- fold for MMP-9, *p* < 0.05) by EGCG (50 μM) treatment.

## 3. Experimental 

### 3.1. Animals

The experimental procedures were approved by the Institutional Animal Care and Use Committee of Samil Pharmaceuticals co., ltd. and conform to the guidelines established by the Korea Food and Drug Administration (KFDA) for the Care and Use of Animals for Scientific Purposes. BALB/c mice and Sprague-Dawley (SD) rats were obtained from Orient bio (Seongnam, Korea). All animals were housed in cages under automatically managed air-conditioned room (23 ± 2 °C and ~60% humidity) and lighting (12-h light–dark cycle).

### 3.2. Cell Cultures

The human RPE cell line ARPE-19 (American Type Culture Collection, Manassas, VA, USA) was cultured in Dulbecco’s modified Eagle’s medium (DMEM) supplemented with 10% fetal bovine serum (FBS), 100 μg/mL penicillin, and 100 μg/mL streptomycin. DMEM, FBS, penicillin and streptomycin were purchased from Gibco (Carlsbad, CA, USA). HRMECs (Cell systems; Kirkland, WA, USA) were cultured in complete medium according to the instructions provided by the manufacturer. These cells were maintained at 37 °C and 5% CO_2_ in a humid atmosphere. ARPE-19 cells and HRMECs used in this study were taken from passages four to seven. In all experiments, dimethyl sulfoxide (DMSO, Amresco, Solon, OH, USA) was added to below 0.01% as a negative control. EGCG (TEAVIGO™, DSM Nutritional Products Ltd, Basel, Switzerland, >98% purity) stock solution was freshly prepared in culture medium or saline after dissolving in DMSO, which was then stored at −20 °C.

### 3.3. Cell Viability

To determine whether EGCG induces a cytotoxic effect in HRMECs and ARPE-19 cells, cell viability was measured by a 3-(4,5-dimethylthiazol-2-yl)-2,5-diphenyltetrazolium bromide (MTT, Calbiochem, San Diego, CA, USA) assay. Cells were seeded in 96-well plates at 5 × 10^3^ cells per well. After 24 h, cells were treated with 1–100 μM of EGCG for 24 h. Next, 10 μL of MTT solution (0.5 mg/mL) was added per each well. After 4 h of incubation, 100 μL of DMSO was added to each treated well and mixed thoroughly by shaking to solubilize the MTT formazan crystals. Absorbance was read on a multiwell scanning microplate reader (Spectra Max 190, Molecular Devices, Sunnyvale, CA, USA) at 570 nm.

### 3.4. Gelatin Zymography on ARPE-19 Cells 

ARPE-19 cells were seeded in 6-well plates at 2 × 10^5^ cells per well. Culture medium was collected 24 h after treatment (10 ng/mL for TPA (Sigma Chemical Co., St Louis, MO, USA), TNF-α (Invitrogen, CA, USA), or 1–50 μM for EGCG) and centrifuged 20 min at 15,000 g at 4 °C. At the time the supernatant was collected, protein quantification was measured by Bradford assay (Bio-Rad, Hercules, CA, USA). 10 μg of protein extracts from each experimental condition were assessed using 10% zymography gels. After electrophoresis, gels were incubated in 2.5% Triton X-100 for 30 min, localized to the 50 mM Tris buffer for 18 h at 37 °C, and incubated for 30 min with 0.1% Coomassie brilliant blue R-250 dye (Bio-Rad, Richmond, CA, USA) for staining target protein. Transparent protein band was detected for zymography against a blue background. The molecular masses (kDa) were identified. All experiments were repeated at least three times, and similar results were obtained.

### 3.5. RNA Isolation and Quantitative Real-Time PCR 

Total RNA was isolated from ARPE-19 cell using the RNeasy Plus mini kit (Qiagen, Valencia, CA, USA) according to the manufacturer’s instructions. RNA purity was confirmed by a 260/280 nm absorbance ratio greater than 1.8. First strand cDNA synthesis was performed by reverse transcription with 1 μg total RNA using the Transcriptor First Strand cDNA Synthesis Kit (Roche Diagnostics. Mannheim, Germany). The synthesized cDNA was used as a template to estimate the quantity of gene transcription by real-time PCR. Specific primers (Bioneer, Daejeon, Korea) and Universal Probe Library (UPL, Roche) were designed and auto-made by Roche Diagnostics. The amplication reactions were performed on a Roche light cycler 480 instrument (Roche Diagnostics) by using Lightcycler^®^ 480 probes master (Roche Diagnostics) and specific primers. The amplification conditions are as follows: 95 °C (10 s), 60 °C (30 s), and 72 °C (1 s) for 45 cycles. The primers and probes for specific genes or glyceraldehyde-3-phosphate dehydrogenase (GAPDH) are listed in Table 1. The relative quantitation was calculated as 2^−∆Ct^ (∆Ct = Ct of the target gene minus Ct of GAPDH).

**Table 1:** Designed primers for quantitative real-time PCR.

Gene	Primer	Sequence
MMP-9	F	5'-GAG TGT AAC CAT AGC GGT ACA GG-3'
	R	5'-TCT TCC CTG GAG ACC TGA GA-3'
VEGFR-1	F	5'-CCA CTC CCT TGA ACA CGA G-3'
	R	5'-GTC GCC TTA CGG AAG CTC-3'
VEGFR-2	F	5'-GAA CAT TTG GGA AAT CTC TTG C-3'
	R	5'-CGG AAG AAC AAT GTA GTC TTT G-3'
GAPDH	F	5'-CCC CGG TTT CTA TAA ATT GAG C-3'
	R	5'-CAC CTT CCC CAT GGT GTC T-3'

F: forward, R: reverse.

### 3.6. Detection of MMP-9 Expression Levels on TPA-Induced ARPE-19 Cells

ARPE-19 cells were seeded in 6-well plates at 3 × 10^5^ cells per well. After 24 h, cells were treated with 10 ng/mL TPA or 1–50 μM EGCG. After incubation for 24 h, total protein and RNA were isolated from each sample. The mRNA expression of MMP-9 was analyzed by the quantitative real-time PCR method as mentioned above. The protein concentrations of MMP-9 were measured using a commercial ELISA kit (Quantikine, R&D Systems, Minneapolis, MN, USA) according to the manufacturer’s protocol. The ELISA kits detect free MMP-9 within the sampled whole-cell lysates to give a value for the MMP-9 activity. 

### 3.7. Cell Viability on H_2_O_2_-Induced ARPE-19 Cells

ARPE-19 cells were seeded in 96-well plates at 2 × 10^4^ cells per well. After 24 h, cells were treated with 600 μM H_2_O_2_ or 1–50 μM EGCG. After incubation for 24 h, cell viability was measured by MTT assay. Absorbance was read on a multiwell scanning microplate reader at 570 nm. 

### 3.8. Intracellular Reactive Oxygen Species (ROS) Assay on ARPE-19 Cells

ROS activity was quantified using an Oxiselect intracellular ROS assay kit (Cell Biolabs, San Diego, CA, USA) according to a modified protocol. Briefly, the ARPE-19 cells were seeded in 96-well plates (2 × 10^4^ cells per well). After 24 h, cells were pretreated with EGCG (1–50 μM) for 30 min and then left untreated or exposed to 600 μM H_2_O_2_ for 15 min. Intracellular ROS level was detected measuring the conversion of 10 µM 2',7'-dichlorofluorescein diacetate (DCF-DA). Fluorescence intensity was determined using VICTOR X2 Multilabel Plate Reader (Perkin-Elmer, Heidelberg, Germany) at 485 nm excitation and 535 nm emission, respectively.

### 3.9. Detection of MMP-9, VEGF, and VEGFR-2 Expression Levels on H_2_O_2_-Induced ARPE-19 Cells

ARPE-19 cells were seeded in 6-well plates at 3 × 10^5^ cells per well. After 24 h, cells were treated with 300 μM H_2_O_2_ or 1–50 μM EGCG. After incubation for 18 h, total RNA was isolated from each sample, and then mRNA expression levels of MMP-9, VEGF and VEGFR-2 were analyzed by the quantitative real-time PCR method as mentioned above.

### 3.10. VEGF-Induced Proliferation Assay

HRMECs were seeded in 96-well plates at 1.2 × 10^4^ cells per well. After 24 h, cells were treated with 10 ng/mL VEGF (R&D Systems) or 1–50 μM EGCG. After incubation for 72 h, cell proliferation activity was measured by MTT assay as mentioned above. 

### 3.11. VEGF-Induced Tube Formation Assay

96-well plates were coated with thin layers of growth factor-reduced Matrigel (BD Labware, Bedford, MA, USA) for 1 h at 37 °C. The HRMECs were seeded in Matrigel coated plates at 5 × 10^3^ cells per well. After 3 h, cells were treated with 10 ng/mL VEGF or 1–50 μM EGCG. After incubation for 24 h, the medium was removed and exchanged. Images were captured through an Eclipse Ti-U fluorescent microscope (Nikon Corp., Tokyo, Japan) at a magnification of 40×. These were then scanned, and images were captured by using NIS BR 3.07 software (Nikon). Tube formation was quantified by counting the length of the tubular polymerization in entire fields at a magnification of 40×. The basal tubular network was measured and subtracted.

### 3.12. In Vitro Vascular Permeability on HRMECs

Vascular permeability on HRMECs monolayer was measured using a vascular permeability assay kit (ECM644, Millipore, Billerica, MA, USA) according to the manufacturer’s protocol. Briefly, HRMECs were seeded into the insert of 24-well plates at 1 × 10^5^ cells per well. After incubation for 72 h, cells were treated with 10 ng/mL VEGF or 1–50 μM EGCG. After 24 h, FITC-dextran working solution (1/40 dilution) was added to each insert, incubated for 20 min at room temperature, in the dark, and then transferred to the reading plate 100 μL permeates from each well of the receiver tray. Finally, the plate was detected using a VICTOR X2 Multilabel Plate Reader (Perkin-Elmer) at 485 nm and 535 nm excitation and emission, respectively.

### 3.13. Miles Assay for in Vivo Permeability

The miles assay was conducted in the back skin of male BALB/c mice (age, 8 weeks). The shaved back skin of mice was prepared 72 h prior to the experiment. Mice were pre-administered orally with EGCG (200 mg/kg) or saline. After 1.5 h, mice were anesthetized by intraperitoneal injection of 1% pentobarbital sodium (nembutal; 60 mg/kg). Next, mice were injected intravenously (tail vein) with 1% Evans Blue dye (Sigma). After 10 min, 50 ng VEGF or saline in 50 µL were injected intradermally into the shaved back area. The mice were sacrificed by cervical dislocation 30 min later, and the entire administered site of skin was obtained. The Evans blue dye was extracted from the skin by using the formamide for 48 h at 60 °C, and the absorbance of the extracted Evans Blue dye was read on a multiwell scanning microplate reader (Molecular Devices) at 620 nm.

### 3.14. Retinal Vascular Permeability (RVP) Assay

The RVP assay was conducted in the eyes of male SD rats (age 7 weeks). Rats were administered orally with EGCG (200 mg/kg) or saline for 4 days. On days 5, rats were anesthetized by intraperitoneal injection of 1% pentobarbital sodium (nembutal; 60 mg/kg). Next, 50 ng VEGF or saline in 5 µL was injected intravitreally into each eye (Right: VEGF and Left: saline). After 10 min, rats were injected intravenously (tail vein) with 1% Evans Blue dye. After 2 h, the chest cavity was opened, and rats were perfused via the left ventricle at 37 °C with PBS (with heparin 21 U/mL) and 4% paraformaldehyde, respectively. Immediately after perfusion, both eyes were directly enucleated, and then were fixed with 4% paraformaldehyde for 1 h. The Evans Blue dye was extracted from both eyes by using the formamide for 48 h at 60 °C, and the absorbance of the extracted Evans Blue dye was read on a multiwell scanning microplate reader (Molecular Devices) at 620 nm.

### 3.15. Corneal Neovascularization (CNV) Assay & Immunohistochemistry (IHC)

The CNV assay was conducted in the eyes of male BALB/c mice (age 7 weeks) by alkali burns. After general anesthesia using a mixture of Zoletil 50^®^ (zolazepam and tiletamine; Virbac Laboratories, Carros, France) and Rompun^®^ (xylazine; Bayer, Seoul, Korea) solution (3:1 ratio, 1 mL/kg, intraperitoneally), 0.5 N NaOH was permeated a 5 mm diameter circular filter disc for 60 s. The filter disc was put on the corneal center for 60 s in anesthetized mice. Immediately after alkali burns, eyes were washed with saline for 30 s and then injected subconjunctivally with EGCG (50 μM, 2 μL) or saline. Corneal photographs were taken with a 25× magnification using a digital camera attached to the slit-lamp microscope (Eclipse Ti, Nikon) on the day 3. After the image capture, both eyes were directly enucleated. Tissue samples were washed rigorously in 0.5% Triton-X 100 in PBS. Before storing in liquid nitrogen (LN_2_), OCT media were used for tissue embedding. Corneal tissue sections (6 μm) were sliced by using a cryomicrotome (CM3050S, Leica, Heidelberg, Germany) and mounted in a standard microscope slide. A microscope specimen was stored at −80 °C until further observation. Fluorescent immunohistochemistry was conducted with a monoclonal antibody to anti-VEGF, anti-CD31, and anti-MMP-9 (1:200 dilution; Santa Cruz Biotechnology, Inc.). Goat anti-rabbit IgG-Rhodamin-conjugated (red) and Goat anti-mouse IgG-Alexa 488 conjugated (green) were used for secondary antibodies (Abcam). Samples were thawed and blocked in 0.5% Triton-X100 in PBS for 10 min. Primary antibodies response to the corneal tissue of overnight incubation in a cold room at 4 °C. Corneal tissues were washed with 0.5% Triton-X100 in PBS for 10 min and incubated in the secondary antibody at room-temperature for 1 h. Tissue sections were washed with 0.5% Triton-X100 in PBS and mounted with Aquamount (Merck). The samples were observed and photographed by using a fluorescence microscope (Carl Zeiss, Axio Imager, Jena, Germany). Double-labeled fluorescent intensities of CD31 and MMP-9 were analyzed by NIH image J software (ver 1.45).

### 3.16. Statistical Analysis

All statistical analyses were performed with SPSS software version 12.0 (SPSS Inc., Chicago, IL, USA). Values were expressed as means ± SD of independent experiments. Statistical significance for differences between the groups were performed using the unpaired Student’s *t*-test and one-way ANOVA. Data with a *p* value less than 0.05 were considered statistically significant.

## 4. Conclusions 

In the present study, we have demonstrated that EGCG effectively protects HRPECs from cell death and attenuated mRNA expressions of MMP-9, VEGF, and VEGFR-2 by ROS-induced oxidative stress. EGCG significantly inhibited proliferation, vascular permeability, and tube formation in VEGF-induced HRMECs. Furthermore, EGCG significantly reduced vascular leakage and permeability in VEGF-induced animal models. In addition, EGCG effectively limited upregulation of MMP-9 and CD31 on CNV model induced by alkaline burn. Importantly, our results suggest that MMP-9 and VEGF is one of the noteworthy therapeutic targets of EGCG for treatment and prevention of ocular angiogenic diseases, such as age-related macular degeneration, diabetic retinopathy, and corneal neovascularization.
